# Assessment of 3.0 Tesla magnetic resonance imaging in Madelung’s deformity: findings and implications

**DOI:** 10.1186/s12891-024-07245-z

**Published:** 2024-02-12

**Authors:** Yimin Ma, Zhe Guo, Ling Wang, Qianqian Wang, Xiaoguang Cheng, Dong Yan

**Affiliations:** 1 Department of Radiology, Beijing Jishuitan Hospital, Capital Medical University, Beijing, China; 2 Beijing Research Institute of Traumatology and Orthopaedics, Beijing Jishuitan Hospital, Capital Medical University, Beijing, China

**Keywords:** Madelung’s deformity, Vickers ligament, Radiotriquetral ligament, Central disc morphology change

## Abstract

**Objective:**

The aim of the study was to investigate the 3.0 Tesla magnetic resonance imaging (MRI) features of Madelung’s deformity.

**Materials and methods:**

The wrist MRI scans of 19 patients clinically diagnosed with Madelung’s deformity and 20 patients without deformity were consecutively selected from Beijing Jishuitan Hospital between April 2019 and December 2022 for observation, in the case group and control group, respectively. Multiple linear regression was used to analyze the factors affecting tilting angle and width of central disc (CD, also termed as triangular fibrocartilage, the main component of triangular fibrocartilage complex), while the chi-square test was used to compare the occurrences of CD (radial) attachment displacement, VL, and RTL. *p* < 0.05 indicated statistical significance.

**Results:**

Madelung’s deformity significantly contributed to the tilting and thickening of the CD. In the case group, the tilting angle and thickness of CD were (51.46 ± 1.33)° and (0.23 ± 0.01) cm, respectively, which was statistically significant (*p* < 0.05); the radial attachment of the CD significantly shifted away from the distal articular surface level (χ^2^ = 39.00, *p* < 0.001), with a mean displacement of (0.97 ± 0.38) cm. Furthermore, the cases demonstrated abnormally developed Vickers ligament (χ^2^ = 35.19, *p* < 0.001) and radiotriquetral ligament (χ^2^ = 25.66, *p* < 0.001).

**Conclusion:**

MRI provides a notable advantage in diagnosing Madelung’s deformity. Compared with the control group, patients with Madelung's deformity exhibited tilting and thickening of the CD. Additionally, the radial attachment of the CD was significantly shifted proximally with abnormal development of Vickers and radiotriquetral ligaments.

## Introduction

Madelung’s deformity was first described by Otto Madelung in 1878 [1]. The current understanding of its etiology remains incomplete. The irregular development of the volar-ulnar aspect of the distal radial epiphysis is responsible for Madelung’s deformity, which results in a shortened radius, dorsal displacement of the distal ulna, and a tapered row of carpal bones [2–4]. Patients may experience pain, reduced strength, and limited mobility [5].

Plain X-ray film and computed tomography (CT) are currently the prevalent diagnostic methods. However, they have many limitations. First, any condition that restricts the development of the volar-ulnar aspect of distal radius epiphysis can result in wrist deformities similar to the Madelung’s (known as Madelung-like deformities). These conditions include epiphyseal injury, tumors, metabolic bone diseases, and osteochondrodysplasia [6, 7]. It can be challenging to differentiate these conditions from true Madelung’s deformity through X-ray examinations. Second, Madelung’s deformity may be linked to the presence of abnormally developed ligaments, including the Vickers ligament (VL) as reported by Vickers and Nielsen et al. in 1992 [8], and the radiotriquetral ligament (RTL) as reported by Stehling and Langer et al. in 2009 [9]. These ligaments cannot be observed via X-ray examinations. Additionally, it has been suggested that the reliability and repeatability of X-ray examination results may be influenced by different measurement methods [2, 10].

The magnetic resonance imaging (MRI) technique offers a superior soft-tissue resolution, without exposing the patient to ionizing radiation. This makes it crucial in both the diagnosis and differential diagnosis of Madelung’s deformity. In this study, we reviewed the 3.0 T MRI images of the wrists of 19 patients clinically diagnosed with Madelung’s deformity, aiming to elaborate the specific MRI features.

## Materials and methods

### Sample selection of the case group

The MRI data of the affected wrists of patients clinically diagnosed with Madelung’s deformity between April 2019 and December 2022 at Beijing Jishuitan Hospital were retrospectively collected, and cases were consecutively selected as to create the case group. The exclusion criteria were as follows: (1) participants with motion artifacts that significantly affected image evaluation and (2) patients whose image evaluation was impacted by anatomic changes or significant implant artifacts resulting from previous wrist surgery.

A total of 19 female participants were enrolled in the case group, aged 9–34 years, with an average age of (15.05 ± 6.63) years.

### Sample selection of the control group

The MRI data of 20 participants with normal wrist morphology at Beijing Jishuitan Hospital since April 2019 were retrospectively and consecutively collected to form the control group. The exclusion criteria were as follows: (1) wrist deformities resulting from trauma, surgery or developmental factors; (2) triangular fibrocartilage complex injury owing to trauma or degenerative changes; (3) volar raiocarpal ligaments wear and tear caused by trauma; and (4) patients whose image evaluation was significantly affected by motion artifacts or postsurgical implant artifacts.

The 20 participants enrolled in the control group were aged 24–39 years, with an average age of 32.00 ± 4.24 years. Among them, 11 were male and aged 24–39 years, with an average age of 32.00 ± 5.08 years; and 9 were female, aged 27–37 years, with an average age of 32.00 ± 3.24 years.

### MRI scanning conditions and parameters

The wrists of the participants underwent scanning using a 3.0 T MRI scanner (Ingenia 3.0 T, Philips Medical Systems, Best, The Netherlands). The patients were placed in prone position and the examined arms were raised above the head, with the palm placed flat downwards (termed as the ‘SUPERMAN’ position). An original manufacturer’s 8-channel wrist coil was employed to guarantee complete wrist coverage. The long axis of the upper arm was aligned as parallel as possible to the direction of the main magnetic field. The scanning parameters of the observed sequences used in this study are listed below:

1. Coronal T1-weighted image (T1WI): repetition time/echo time: 480 ms/15 ms, slice thickness: 2.0 mm; flip angle: 90 degrees; imaging matrix: 292 × 208.
2. Coronal proton density-weighted image with fat suppression (PDWI-FS): 2600 ms/35 ms, slice thickness: 2.0 mm; flip angle: 110 degrees; imaging matrix: 292 × 253.
3. Sagittal PDWI-FS image: 2600 ms/35 ms, slice thickness: 2.6 mm; flip angle: 110 degrees; imaging matrix: 360 × 168.
4. Axial PDWI-FS image: 2600 ms/35 ms, slice thickness: 2.0 mm; flip angle: 90 degrees; imaging matrix: 268 × 215.

### Measurement and analysis of images

**Fig. 1:**
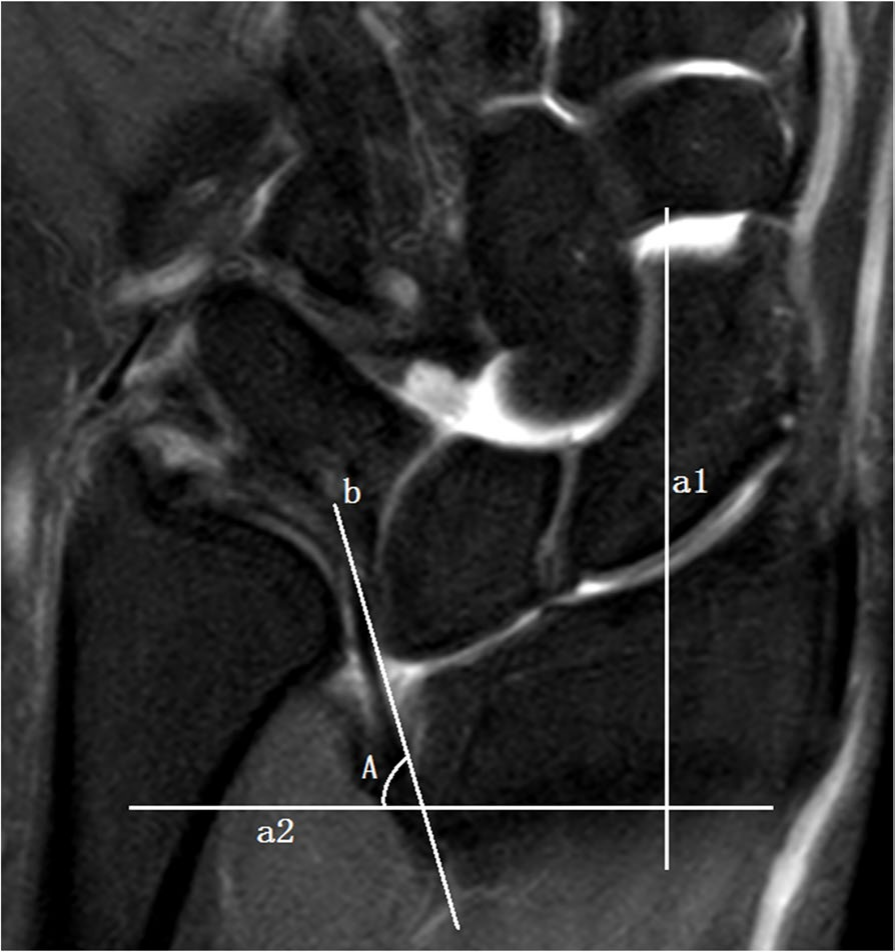
Measurement of CD tilting angle. The perpendicular line (a2) to the long axis of the radius (a1) was drawn, and it intersected the long axis of the radial attachment of the CD (b) to form an acute angle ∠A as the tilting angle, which was used to evaluate the tilting degree of the CD. (Example image No. 1018****)

**Fig. 2:**
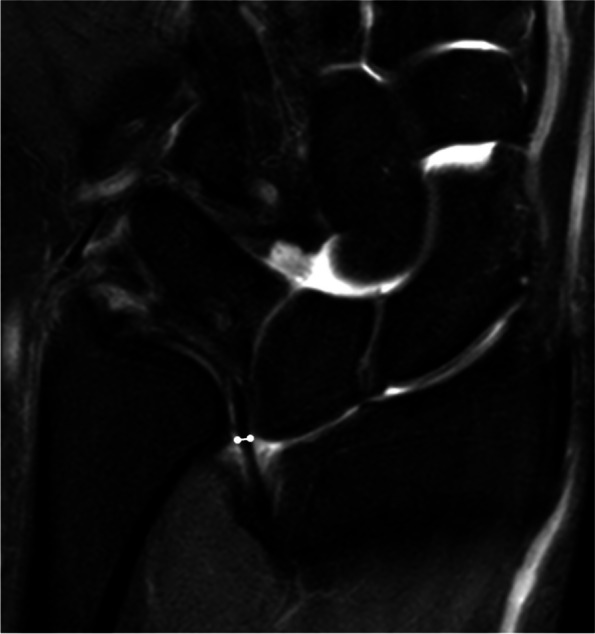
Measurement of CD minimal thickness. The image through the middle plane of CD was selected to measure the thickness of the narrowest part of CD (white line segment). (Example image No. 1018****)

**Fig. 3:**
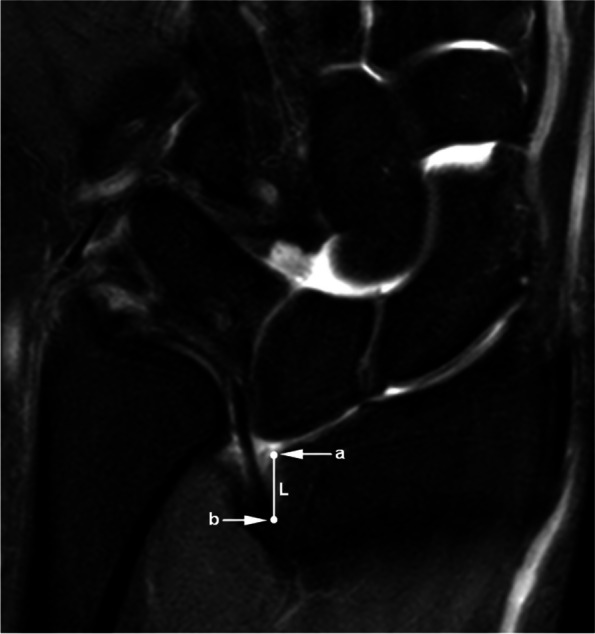
Measurement of CD attachment displacement: The length of the line segment (L) between the medial rim of distal radial articular surface (point a) and the radial attachment of CD (point b) was recorded as the attachment displacement, demonstrating the proximal shift distance of CD insertion. (Example image No. 1018****)

The wrist MRI images of the participants were collected and reviewed by two experienced senior musculoskeletal radiologists who were blinded to the clinical diagnosis. All images were measured using the following indicators:

1. Tilting angle of central disc (CD, the main component of triangular fibrocartilage complex): The coronal T1WI and/or PDWI-FS images through the middle plane of CD were selected. A perpendicular line to the long axis of the radius was drawn, and the angle between the long axis of CD and the aforementioned perpendicular line was measured to evaluate the tilting degree of CD (Fig. 1).
2. Width of CD: The coronal T1WI and/or PDWI-FS images through the middle plane of CD were selected to measure the thickness of the narrowest region of the CD (Fig. 2).
3. Displacement of CD radial attachment: The coronal T1WI and/or PDWI-FS images through the middle plane of CD were selected to measure the shifted distance between the CD radial attachment and the medial rim of distal radial articular surface (Fig. 3). If the distance was greater than 0, it was recorded as displacement positive ( +); if the distal surface of CD continued with the hyaline cartilage covering the articular surface, it was recorded as displacement negative (-).
4. VL evaluation: If VL was observed, it was recorded as positive ( +), and the coronal or sagittal images that could display the maximum cross-sectional area of VL were selected to measure the length and thickness of the ligament respectively; otherwise, it was recorded as negative (-).
5. RTL evaluation: If RTL was observed, it was recorded as positive ( +), and the coronal images showing the maximum cross-sectional area of RTL were selected to measure the length and thickness of the ligament respectively; otherwise, it was recorded as negative (-).

### Statistical analysis

SPSS 26.0 software (IBM SPSS Inc., Chicago, IL, USA) was used for statistical analysis of all data.

The intraclass correlation coefficient (ICC) was used to evaluate the consistency of all quantitative data. The evaluation standards were as follows: ICC > 0.75 indicated good consistency; ICC: 0.50–0.75 indicated general consistency; and ICC < 0.50 indicated poor consistency. The chi-square test was used to evaluate the consistency of all qualitative data. The outcomes showed that the ICC value of RTL width was general (ICC = 0.728, *p* < 0.001), and the consistency of the rest of the quantitative data was good (ICC: 0.777–0.975, *p* < 0.001). Furthermore, all qualitative data showed good consistency (Kappa = 1.000, *p* < 0.001). Therefore, all measurement methods passed the consistency test, and subsequent statistical analyses were conducted using the measurement results from the first radiologist.

The distributions of tilting angle and width of CD were pre-analyzed using Kolmogorov–Smirnov test, confirming that the two followed a normal distribution. The T test was used to assess the differences between groups. The factors affecting tilting angle and width of CD were analyzed by multiple linear regression. The chi-square test was used to compare the occurrences of CD (radial) attachment displacement, VL, and RTL. *p* < 0.05 indicated statistical significance.

## Results

### Statistical results

Multiple linear regression results showed that Madelung’s deformity was a significant positive influencing factor for CD tilting angle (*p* = 0.001) and width (*p* = 0.012) (Table 1), after controlling for age and sex. The CD tilting angle and width of the case group were (51.46 ± 1.33)° and (0.23 ± 0.01) cm respectively, both were significantly different from those of the control group (*p* < 0.05).

**Table 1:** Multiple linear regression analysis of the tilting angle and width of the CD was performed using the measurement data from the first radiologist

Group	Tilting angle of TFC (mean ± SD)		Width of TFC (mean ± SD)	
	Unadjusted	Adjusted^a^	Unadjusted	Adjusted^a^
Control group	26.45 ± 7.37	26.48 ± 2.28	0.15 ± 0.05	0.15 ± 0.01
Case group	51.48 ± 9.78	51.46 ± 1.33	0.23 ± 0.06	0.23 ± 0.01
*P* value	< 0.001	0.001	< 0.001	0.012

^a^Adjusted for sex and age

The results of chi-square test showed that the radial attachment of CD in the case group was shifted significantly away from the medial rim of distal articular surface (*p* < 0.001) (Table 2), with a mean displacement of (0.97 ± 0.38) cm. Compared with the control group, VL was observed in the case group (*p* < 0.001), with an average length of (1.05 ± 0.25) cm and an average width of (0.19 ± 0.05) cm; RTL was observed in the case group (*p* < 0.001), with an average length of (1.58 ± 0.23) cm and an average width of (0.17 ± 0.02) cm.

**Table 2:** Comparisons of (the occurrences of) CD radial insertion displacement, VL and RTL between the case group and control group were performed using the measurement data from the first radiologist. Chi-square test was used for qualitative data analysis

Varialbes	Group		*P* value
	Control	Case	
Attachment displacement (n, %)			
- (No)	20 (100%)	0 (0%)	< 0.001
+ (Yes)	0 (0%)	19 (100%)	
VL (n, %)			
- (No)	20 (0%)	1 (5%)	< 0.001
+ (Yes)	0 (0%)	18 (95%)	
RTL (n, %)			
- (No)	20 (100%)	4 (21%)	< 0.001
+ (Yes)	0 (0%)	15 (79%)	

In this study, seven patients in the case group underwent surgery during which the VL was definitively resected. MRI scans detected VL in all seven patients undergoing surgery.

### Presentation of key imaging data of some cases

**Fig. 4:**
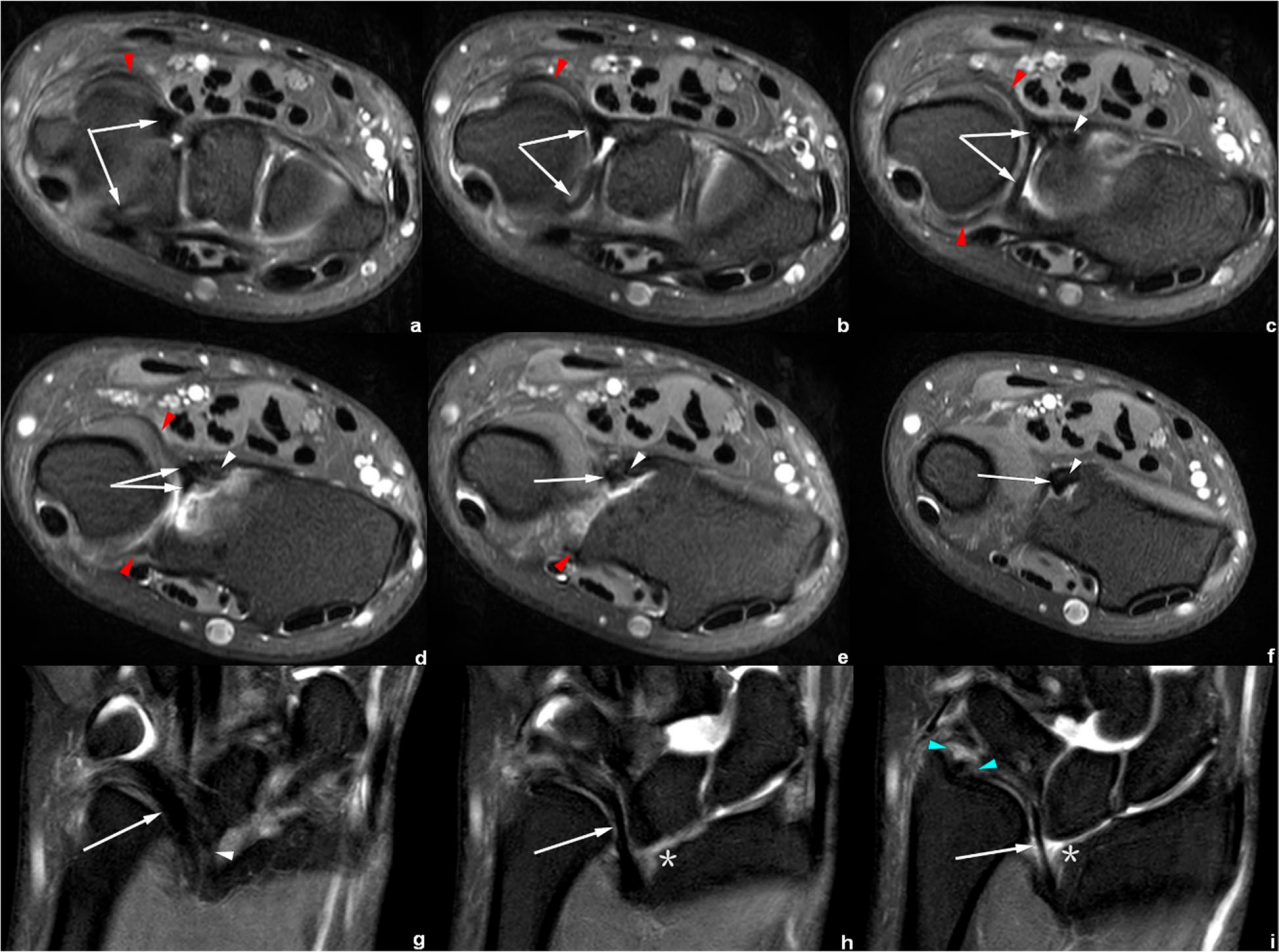
Right wrist MRI of the patient No. 1018****. **a**–**f** Axial PDWI-FS images show the CD (white arrow), volar and distal radioulnar ligament (red arrowhead) and VL (white arrowhead). **g**–**i** Coronal PDWI-FS images show the VL (white arrowhead) and a tilted, thickened CD (white arrow) with the radial insertion significantly shifted proximally from the medial rim of distal radial articular surface (*). The blue arrowheads demonstrate the distal and proximal triangular ligaments, which attached the CD to the distal ulna

**Fig. 5:**
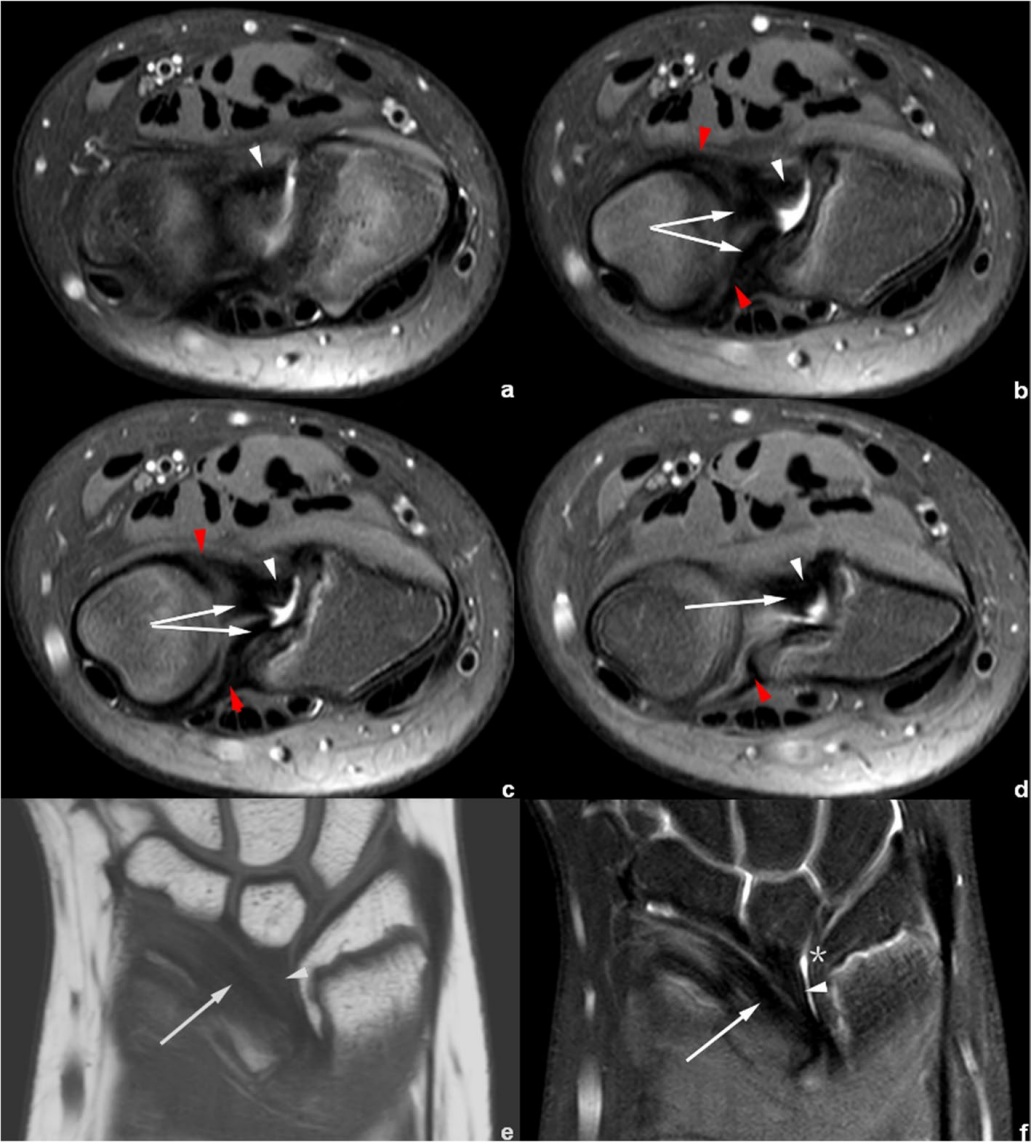
Right wrist MRI of the patient No. 1080****. **a**–**d** Axial PDWI-FS images show the CD (white arrow), volar and distal radioulnar ligament (red arrowhead), and VL (white arrowhead). **e** Coronal T1WI image and **f**) coronal PDWI-FS image show the VL (white arrowhead) and a tilted, thickened CD (white arrow). Notice that the insertion of CD shifted proximally from the medial rim of distal radial articular surface (*)

On CD-related abnormal changes, Figs. 4 and 5 show a tilted and thickened CD with a displacement of radial attachment; these features could be observed in all the cases of Madelung’s deformity in clinical practice.

**Fig. 6:**
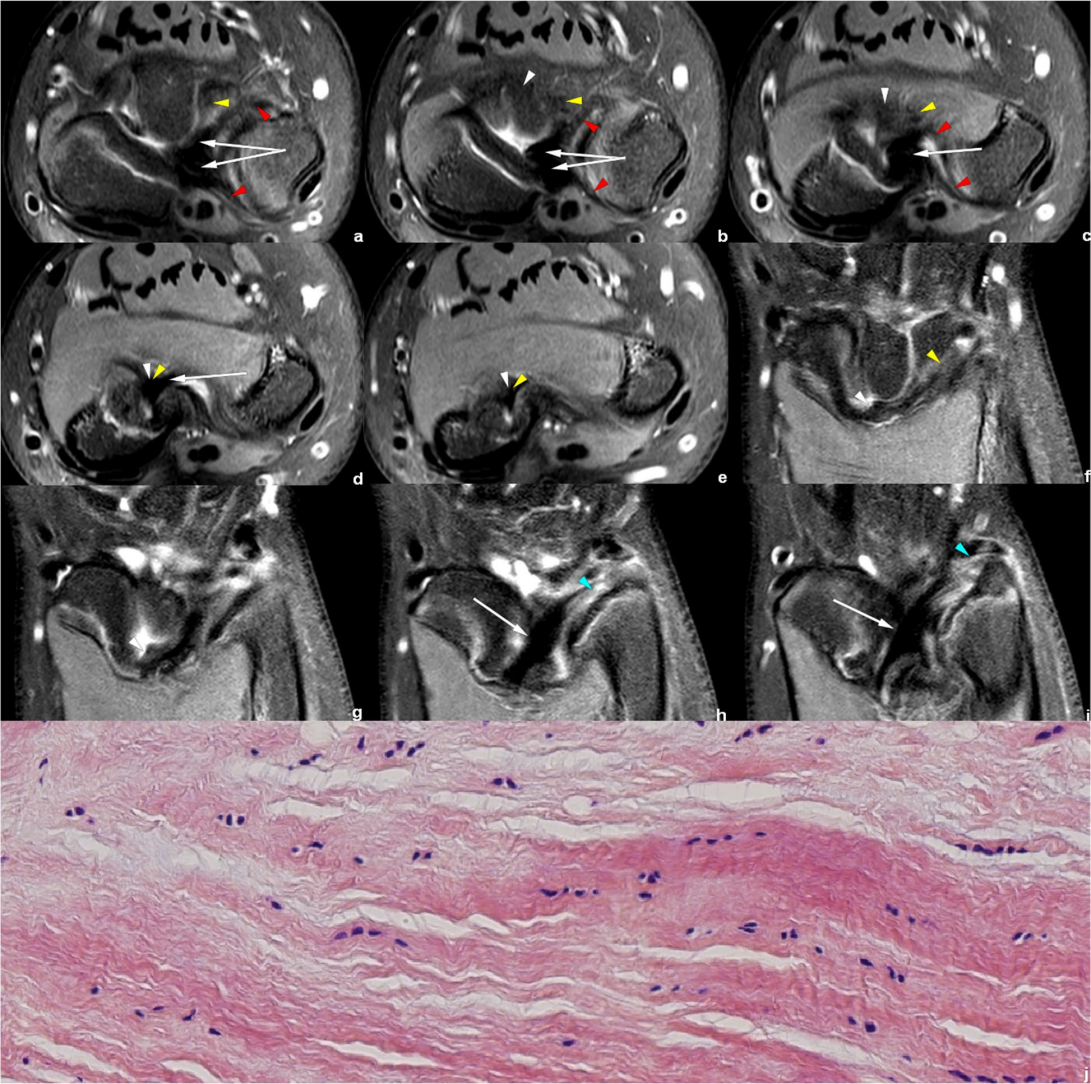
Left wrist MRI of the patient No. 0959****. **a**-**e** Axial PDWI-FS images show the CD (white arrow), volar and distal radioulnar ligament (red arrowhead), VL (white arrowhead), and RTL (yellow arrowhead). **f**–**i** Coronal PDWI-FS images show the VL (white arrowhead), RTL (yellow arrowhead), and CD (white arrow). The blue arrowheads demonstrate the distal and proximal triangular ligaments attaching CD to the distal ulna. This patient underwent surgery to remove the “cord-like tissue connecting the distal radius to the volar aspect of proximal lunate.” **j** Pathological findings show disordered dense fibrous connective tissues and some fat

**Fig. 7:**
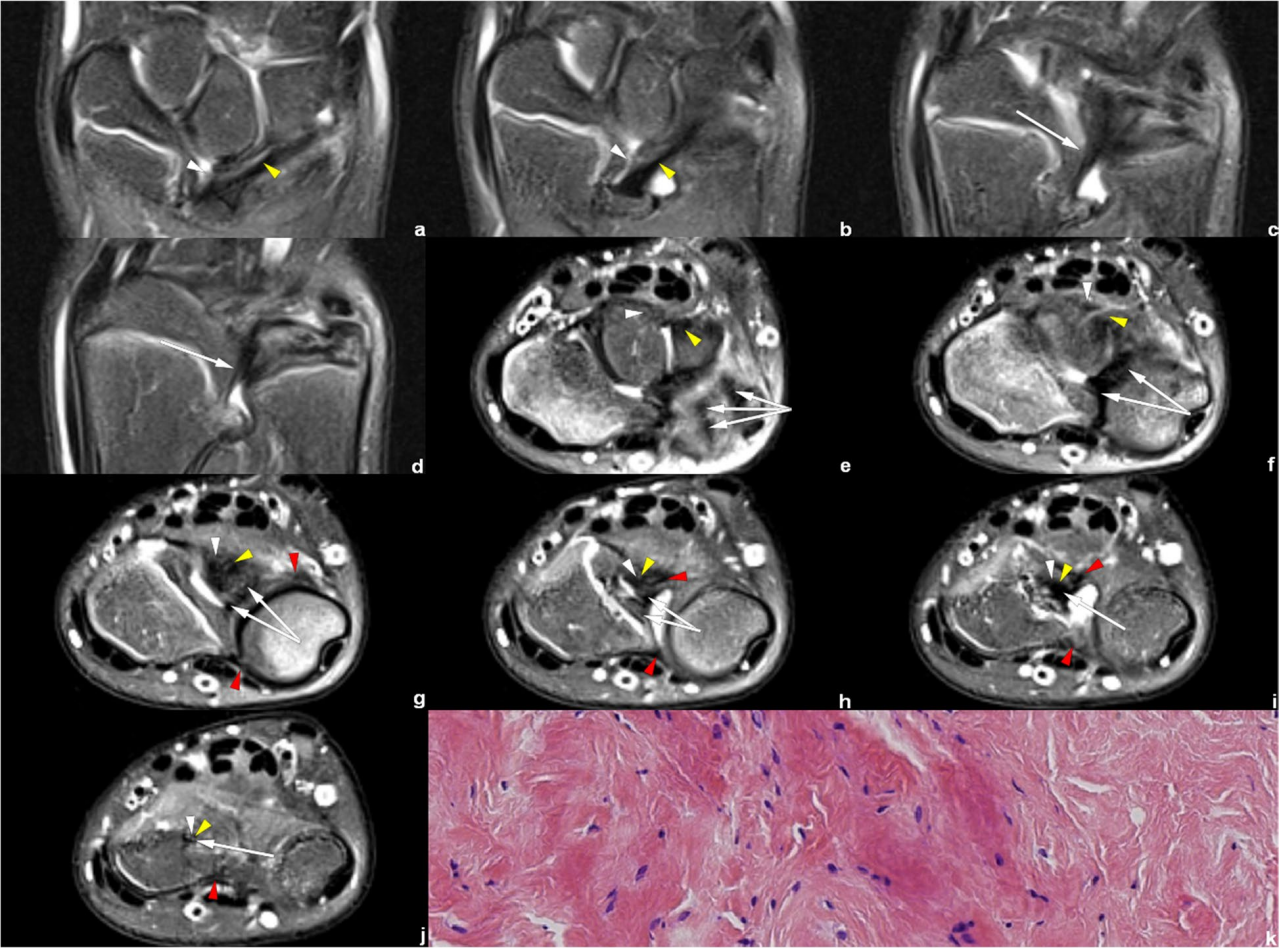
Left wrist MRI of the patient No. 0919****. **a**–**d** Coronal PDWI-FS images show the VL (white arrowhead), RTL (yellow arrowhead), and CD (white arrow). **e**–**j** Axial PDWI-FS images show the CD (white arrow), volar and distal radioulnar ligament (red arrowhead), VL (white arrowhead), and RTL (yellow arrowhead). **k** The postoperative pathological findings show dense fibrous connective tissue

**Fig. 8:**
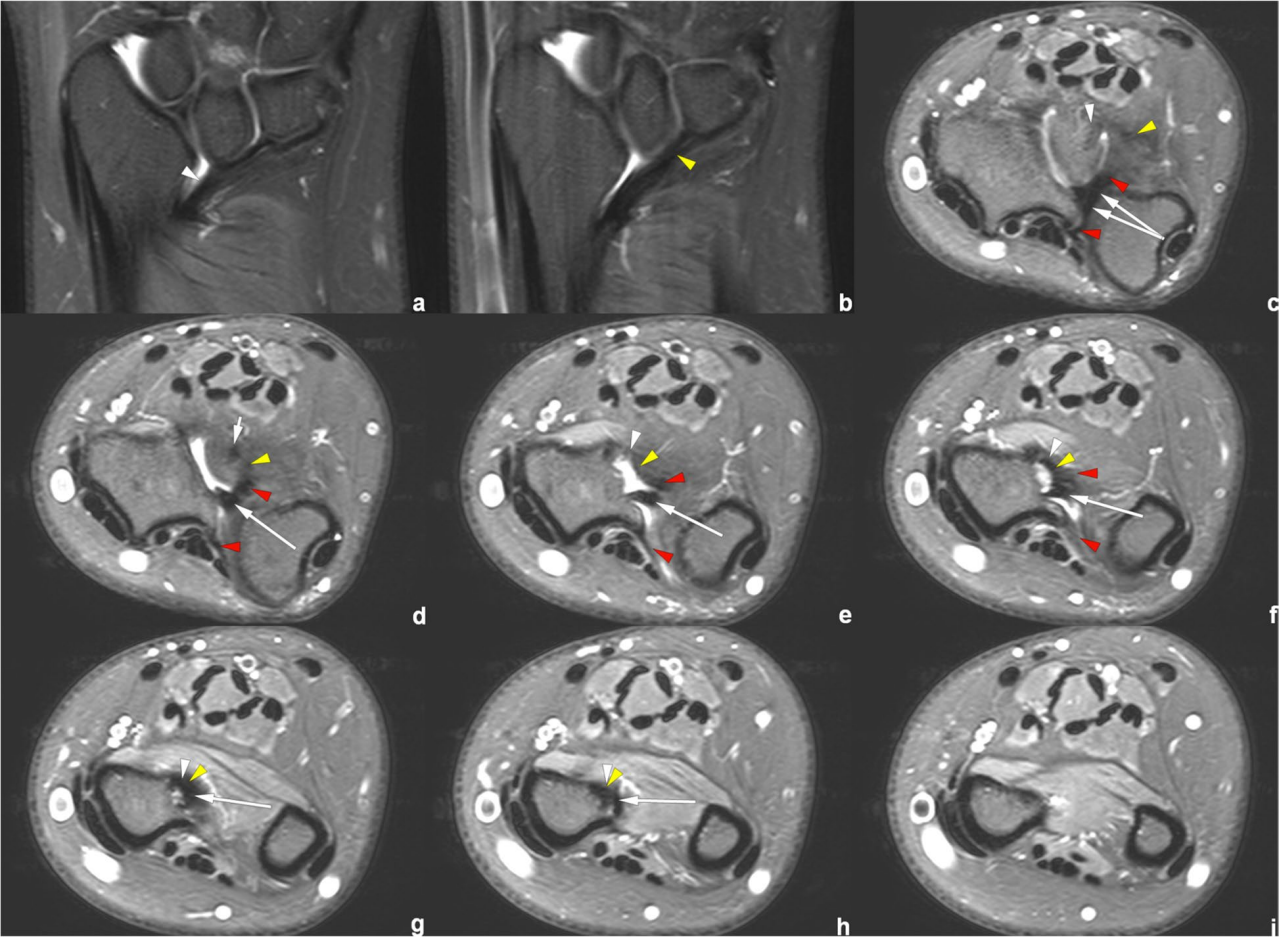
Left wrist MRI of the patient No. 0621****. **a**–**b** Coronal PDWI-FS images show the VL (white arrowhead) and RTL (yellow arrowhead). **c**–**i** Axial PDWI-FS images show the CD (white arrow), volar and distal radioulnar ligament (red arrowhead), VL (white arrowhead), and RTL (yellow arrowhead)

**Fig. 9:**
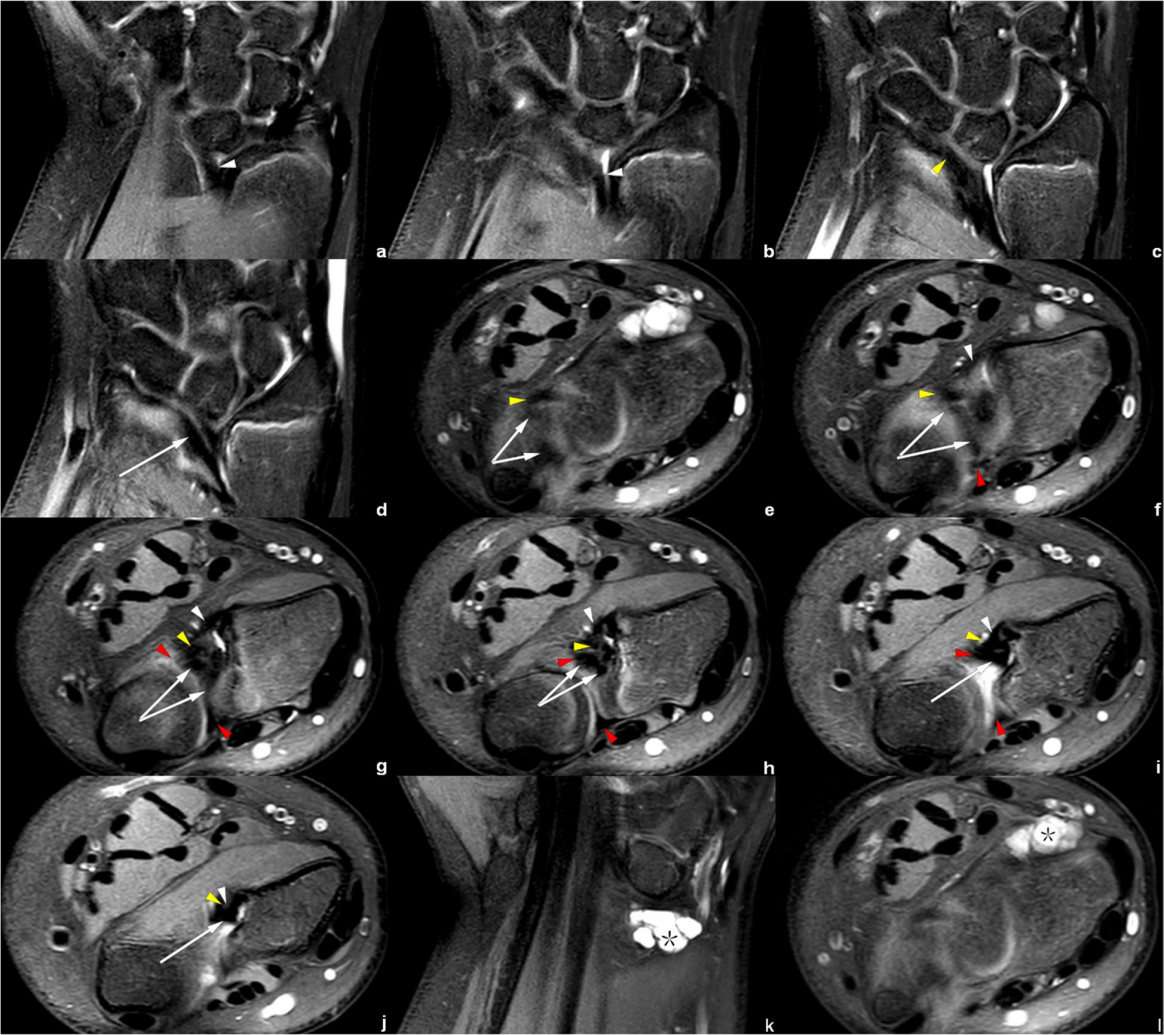
Right wrist MRI of the patient No. 0769****. **a**–**d** Coronal PDWI-FS images show the VL (white arrowhead), RTL (yellow arrowhead), and CD (white arrow). **e**–**j** Axial PDWI-FS images show the CD (white arrow), volar and distal radioulnar ligament (red arrowhead), VL (white arrowhead), and RTL (yellow arrowhead). **k**–**l** Coronal and axial PDWI-FS images show a ganglion cyst. The lesion was detected by accident

Figures 4, 5, 6, 7, 8 and 9 show the abnormally developed VL. Two of the patients (No. 0919**** and No. 0959****) underwent surgery, and the surgical notes indicated “excision of the fibrous band-like structure tethering the distal radius to the volar aspect of proximal lunate”. The attached pathological report, of each patient, demonstrated that the resected structure was dense fibrous connective tissue. Therefore, the resected structures were confirmed to be abnormally developed Vickers ligament.

Figures 6, 7, 8 and 9 show RTL in four cases. We have noticed that the VL and RTL appear to insert at the same site of radius, very close to the insertion of CD.

## Discussion

At present, the cause of Madelung’s deformity remains uncertain. It is believed that the abnormally developed VL forms a band-like structure on the volar-ulnar aspect of the distal radius, which restricts normal growth [8, 11]. When the ligament is released by surgery, the long-term follow-up of patients has shown the maintenance of original radiographic correction, good functional outcomes, and the relief of pain [12, 13]. Many reseaches have suggested that VL is a pathologically thickened short radiolunar ligament [9, 14, 15], with its radial attachment located at the distal metaphysis level [8, 14, 15]. In addition, the RTL reported by Stehling et al. is rare in the literature on other wrist diseases [9]; the incidence, specificity, and function of this ligament need to be further studied [16].

**Fig. 10:**
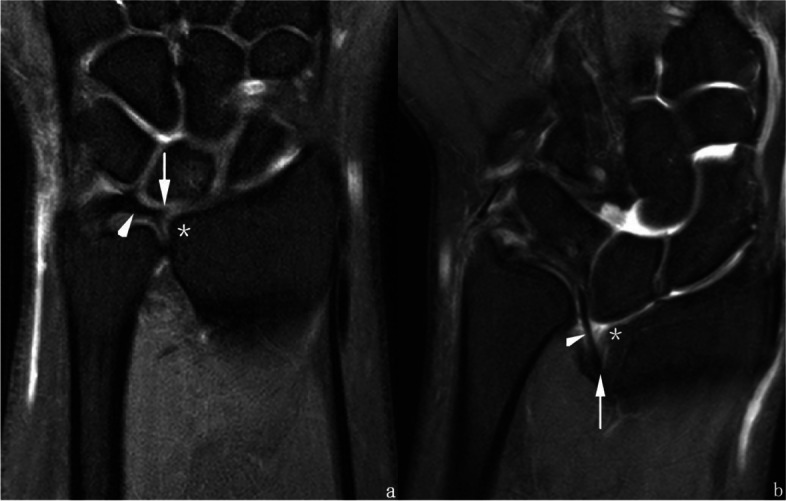
Comparison between **a**) a normal wrist from the control group (patient No. 0478****) and **b**) a wrist with Madelung’s deformity (patient No. 1018****). The coronal PDWI-FS images show the radial insertion (white arrow) of CD (white arrowhead). The normal CD inserted at the radius at the level equal to the distal articular surface (*), while in the Madelung’s deformity image the insertion appears to shift proximally (*)

MRI can help to differentiate Madelung’s deformity from Madelung-like deformities with developed characteristic structures: VL and RTL. These ligaments are not seen in Madelung-like deformities (nor in the normal wrists) and serve to differential diagnosis [15, 16]. Furthermore, many researches have described the tilting and thickening of the CD in patients with Madelung’s deformity [9, 14, 15, 17]; however, the displacement of the CD radial attachment has been rarely demonstrated. Generally, the triangular fibrocartilage complex attaches to the radius via radioulnar ligaments (volar and dorsal) and CD; the radioulnar ligaments insert at bony entheses of the distal radius, while CD transitions into the hyaline cartilage. In our study, we observed that the radial insertion of CD shifted proximally significantly, with a displacement of approximately (0.97 ± 0.38) cm (Fig. 10). This displacement might be caused by the deformity of the epiphysis, which was tethered and “pulled down” proximally by the VL [8, 15].

**Fig. 11:**
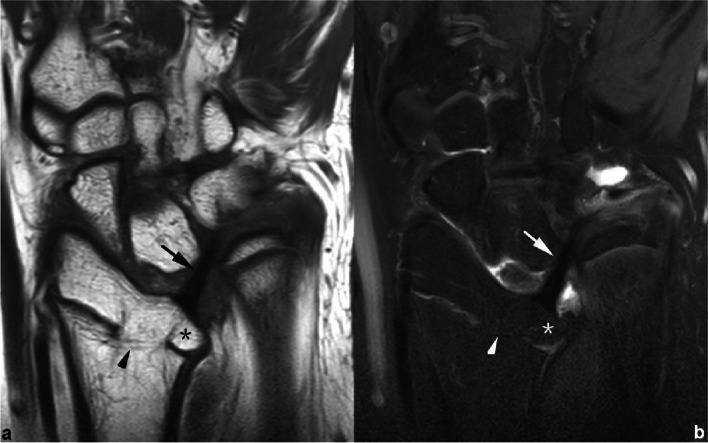
Left wrist MRI of the patient No. 0512****. **a** Coronal T1WI image and **b**) coronal PDWI-FS image show the radial attachment of CD (black/white arrow) was located at the distal articular surface level (black/white *) without proximal displacement. No definite VL and/or RTL were observed in this case. Hence, patient No. 0512**** should be diagnosed with Madelung-like deformity, caused by the local premature closure region (black/white arrowhead) of the distal radial growth plate

**Fig. 12:**
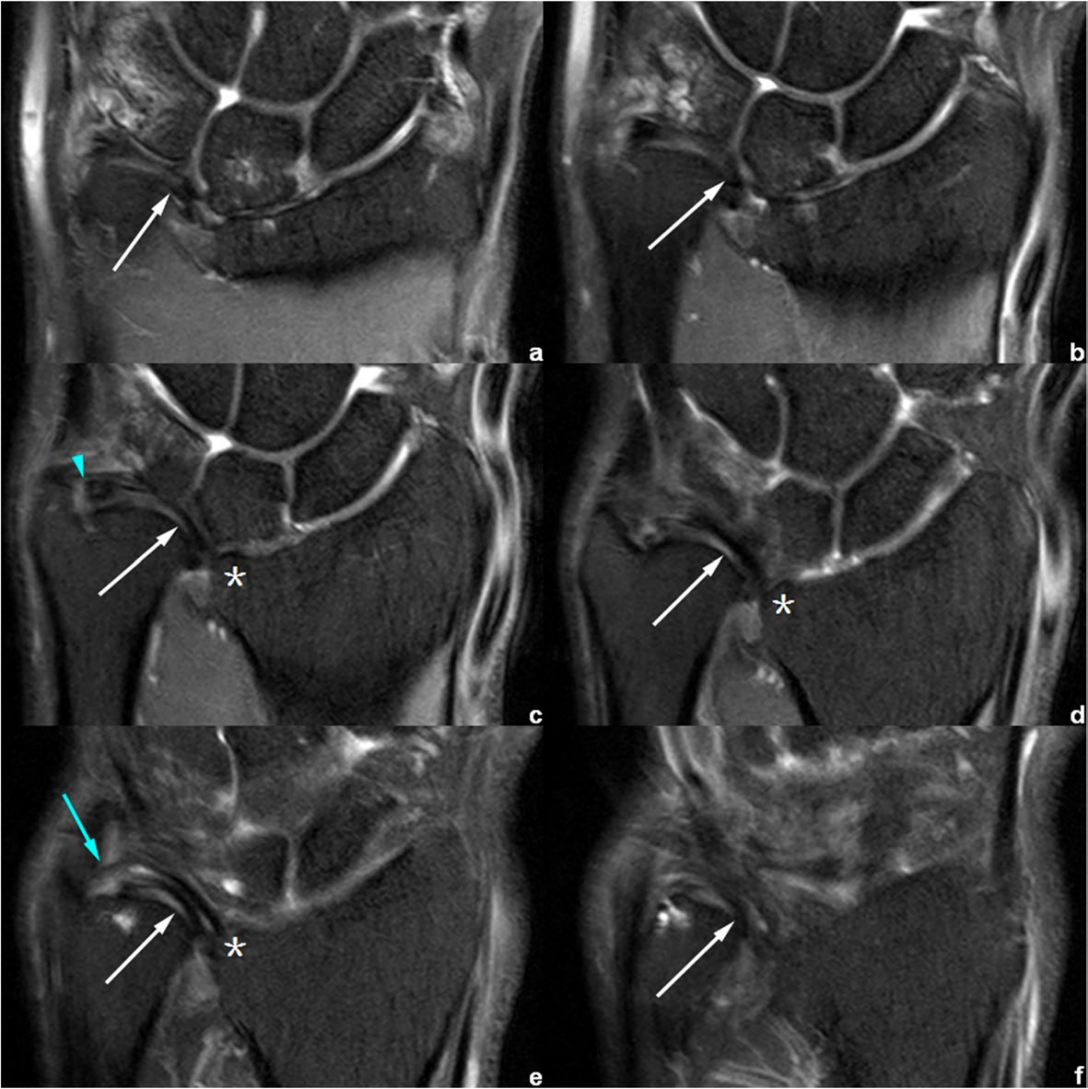
Right wrist MRI of the patient No. 0756****. **a**–**f** Coronal PDWI-FS images show that the CD (white arrow) inserted at the distal articular surface level (*) of radius, and attched to distal ulna via distal (blue arrowhead) and proximal (blue arrow) triangular ligaments. Notice that the CD had degenerative changes due to the abnormal stress. This adult patient had manifestation similar to Madelung’s deformity, but without VL and/or RTL developed. He was diagnosed with Madelung-like deformity of right wrist, according to imaging fingdings and medical history

Although the “displacement” of CD insertion may also occur in Madelung-like deformities with its degree depending on the variable onset time and duration of the primary disease, it presents much more commonly in Madelung’s deformity, since the latter one is a developmental matter having impact on epiphysis at early stage. Thus, a patient who has suffered related carpal rows changes without any displacement of the CD radial insertion should be diagnosed with Madelung-like deformities. This hypothesis was useful in the identification of two cases (neither included in the case group, nor in the control group) in the clinical work (Figs. 11 and 12). In these two patients, little difference occurred in the plain X-rays and CT images depicting the Madelung’s deformity, whereas MRI scans provided key advantages in diagnosis.

The occurrence of VL and RTL plays an important role in diagnosing Madelung’s deformity, but the displacement of CD radial insertion may have its own value. That is, VL and RTL are not always observable due to their thin, flat morphology (especially in 1.5 Tesla MRI images), while CD is relatively easier to display.

In the measurement consistency test, the ICC of RTL width and length was relatively low (0.728 and 0.777, respectively). This was because RTL had a narrower shape and a larger inclination, and it was also susceptible to the partial volume effect within the coronal images. Many difficulties occur regarding the evaluation of anatomical details. Therefore, to provide the most valuable visual evaluation, the radial insertion of ligaments was imaged in the axial plane, as shown in Figs. 4, 5, 6, 7, 8 and 9.

Notably, our findings differ from the existing literature. In many cases, we found VL shares the same insertion with RTL; yet Hanson et al. demonstrated that the radial attachment of RTL is located immediately ulnar and dorsal to VL [16]. In our opinion, the difference may be caused by variations in the RTL insertion point. However, there was neither explicit mention of the RTL insertion at the time of surgery in our investigation, nor studies supporting our findings. Therefore, further research is required including the analysis of more cases to clarify the radial attachment of RTL.

In clinical practice, Madelung’s deformity typically occurs during the second decade of life, while the control group with various wrist joint symptoms were mostly adults, since younger people in their teens seldom suffer wrist discomfort. Furthermore, Madelung’s deformity demonstrates a 4:1 predominance to female [17] and the proportion may be even higher according to our observation. Thus, our “case group” and “control group” have quite different age (9–34 years v.s. 24–39 years, respectively) and sex (19 females:0 males v.s. 9 females:11 males, respectively) distributions. To reduce the impact of the distribution imbalance on the results, we adjusted for age and sex when applying the multiple linear regression model. However, the adjustments could not eliminate the bias completely. In future research, the sample size should be increased, and more effort should be made to match the age and sex distribution between the case and control groups.

Further limitations existed in this study. First, only 19 cases were included, this is a small sample size which could lead to bias in the statistical analysis. Second, the measurement of some key anatomical structures was affected by the small size and irregular shape of the structures, the partial volume effect of scanning, and the subjectivity of the radiologists. Hence, the obtained values may have large errors. Moreover, owing to the small number of cases, we could not determine the observational accuracy of VL and RTL using MRI, nor could we calculate their false positive rate.

In conclusion, this study demonstrated the superiority of MRI in diagnosing Madelung’s deformity. The MRI images found that the CD of these patients was tilted and thickened, and the radial attachment shifted significantly away from the distal articular surface level. Furthermore, patients with Madelung’s deformity had abnormally developed VL and RTL in the wrist. Such features may be used to accurately diagnose and differentiate between Madelung’s deformity and Madelung-like deformities, and add to the current knowledge regarding their etiologies.

## Data Availability

The datasets (acquired from Beijing Jishuitan Hospital, Capital Medical University) analysed during the current study are available from the corresponding author on reasonable request.

## Abbreviations

CD: Central disc
CT: Computed tomography
ICC: Intraclass correlation coefficient
MRI: Magnetic resonance imaging
PDWI-FS: Proton density-weighted image with fat suppression
RTL: Radiotriquetral ligament
T1WI: T1-weighted image
VL: Vickers ligament
